# Combined effects of mixing ratios and tree size: how do mixed forests respond to climate and drought events?

**DOI:** 10.3389/fpls.2024.1477640

**Published:** 2024-10-10

**Authors:** Xiaoxia Wang, Lulu He, Haseen Ullah, Xiaopeng Shi, Jingyu Hou, Yadong Liu, Yang Liu, Liu Xue, Baohua He, Jie Duan

**Affiliations:** ^1^ State Key Laboratory of Efficient Production of Forest Resources, Beijing Forestry University, Beijing, China; ^2^ College of Forestry, Beijing Forestry University, Beijing, China; ^3^ National Energy Research and Development (R&D) Center for Non-food Biomass (NECB), Beijing Forestry University, Beijing, China; ^4^ Key Laboratory for Silviculture and Forest Ecosystem in Arid, and Semi-Arid Region of State Forestry and Grassland Administration, Beijing Forestry University, Beijing, China; ^5^ Resource Conservation Section, Beijing Xishan Experimental Forest Farm Management Office, Beijing, China

**Keywords:** *Pinus tabuliformis*, *Quercus variabilis*, mixing ratios, tree size, climate-growth relationship, drought

## Abstract

Although the relationship between biodiversity and ecosystem functionality (BEF) has been studied comprehensively, how the mixing ratio of tree species in mixed forests affects the response of trees to climate and drought remains an unexplored and rather unknown question. Hence, we established tree-ring chronologies for *Pinus tabuliformis* Carr. (P) and *Quercus variabilis* Blume. (Q) mixed forests with different mixing ratios. In the temperate region of China, we investigated three mixing ratios: 90% P and 10% Q (P9Q1), 60% P and 40% Q (P6Q4), and 20% P and 80% Q (P2Q8). We collected tree ring samples using three tree size categories: dominant, intermediate, and suppressed trees. We explored the climate sensitivity of these trees and their drought tolerance indices–resilience (Rs), resistance (Rt), and recovery (Rc) under two drought conditions: short-term drought (1993 drought) and long-term drought (1999-2015 drought). P6Q4 made *P. tabuliformis* more sensitive to the Palmer drought severity index (PDSI) from the previous year than the other two ratios. The effect of the mixing ratio on drought response was insignificant under short-term drought in both tree species. Rt, Rc, and Rs of *P. tabuliformis* decreased with an increasing *Q. variabilis*:*P. tabuliformis* ratio in long-term drought. Rt, Rc, and Rs of *Q. variabilis* were the highest in P6Q4. The sensitivity of trees to PDSI varied among classes and was influenced by the mixing ratio. Dominant trees were most sensitive to PDSI in P6Q4 and P2Q8, whereas intermediate and suppressed trees were more sensitive to PDSI in P9Q1. The impact of tree size on drought tolerance indices varied according to drought type and mixing ratio. These findings showed that the mixing ratio has a confounding effect on the drought sensitivity of temperate tree species. Differences in hydrological niches allow *Q. variabilis* to benefit from mixing with *P. tabuliformis*. Mixing with optimal proportion of *P. tabuliformis* maximizes the drought resilience of *Q. variabilis*. Additionally, weakly competitive species (*P. tabuliformis*) do not benefit from mixed forests during prolonged water deficits. This result complements previous arguments that species mixing reduces the biological vulnerability of individuals. This study emphasizes the importance of species selection based on the biological and physiological characteristics of tree species in the afforestation of mixed forests. It highlights the critical role of species mixing ratios in the resistance of mixed forest ecosystems to climate change, which may provide a reference for sustainable forest management.

## 1 Introduction

Human-induced climate change has been estimated to have increased temperatures by 0.8°C to 1.2°C above pre-industrial levels (Kang et al., 2021). Concomitantly, climate change increases the incidence of extreme weather events, such as droughts (Charlet De Sauvage et al., 2023). The average air temperature in China has risen by 0.22°C per decade since the 1950s, which is higher than the global average warming rate of 0.13 ± 0.03°C per decade (Shang et al., 2022), and is expected to continue to warm (Wang et al., 2022). A sustained increase in the frequency and intensity of drought events reduces plant growth (Shestakova et al., 2016) and increases mortality risk (Merlin et al., 2015; De Streel et al., 2022). Therefore, it is critical to understand how plants respond to climate change and drought events and how to create appropriate forest management to mitigate these effects (Charlet De Sauvage et al., 2023). Climate sensitivity of trees is often achieved by analyzing the correlation between annual ring width and climate variables (Bello et al., 2019). Three drought tolerance indices can be considered to assess the drought sensitivity of trees: resistance, recovery, and resilience (Grimm and Wissel, 1997; Merlin et al., 2015). Resistance refers to the ability of a plant to maintain an essential state of growth despite drought conditions, recovery refers to the ability to resume growth after a drought, and resilience refers to the ability of a plant to return to pre-drought growth levels after a drought (Merlin et al., 2015; Chen et al., 2023). These metrics have been widely used to quantify plant responses to drought events (Lloret et al., 2011; Chen et al., 2023).

Some studies have indicated that mixed forests exhibit higher productivity and greater resilience to future climate change compared to pure forests (Bastos et al., 2024; Guignabert et al., 2024; Feng et al., 2022; Fuchs et al., 2021; Liu et al., 2022; Steckel et al., 2020; Thurm et al., 2016; Cao et al., 2022). This is because the nutrient cycling efficiency, water efficiency, fine root productivity, and microbial community diversity of mixed forests are higher than those of pure forests (Chen et al., 2023). However, other studies have argued that mixed forests increase competition for resources and are thus more vulnerable to climate change, especially drought events (Fuchs et al., 2021; Nothdurft and Engel, 2020; Jia and Wang, 2024; Liu et al., 2024). Research on the interaction between mixed forests and climate change predominantly focuses on Europe, with tree species such as *Fagus sylvatica* L., *Pinus sylvestris* L., and *Quercus petraea* (Matt.) Liebl., and *Picea abies* (L.) H. Karst (Charlet De Sauvage et al., 2023). In contrast, there is limited research on adapting the temperate mixed forests of China to climate change. Furthermore, determining the appropriate tree species mixture ratio during afforestation is crucial for enhancing stand productivity and stability (Zha et al., 2022). The mixing ratio of tree species not only directly affects the structural composition of the stand but also has a profound impact on the growth and development of the stand by adjusting the functional characteristics of the above- and below-ground organs of the trees (Qin et al., 2016; Zha et al., 2022). Additionally, mixing ratios can produce different adaptive responses to drought conditions by adjusting the microclimate within the stand, such as temperature and humidity, and by regulating the strength and direction of intraspecific and interspecific interactions (Chen et al., 2023). However, there is a scarcity of studies specifically investigating the growth response of different tree species within stands with varying tree species mixture ratios to climate and drought, and this remains an unknown and urgent problem.

In addition to the mixing ratio, diameter class is an important factor influencing the climate sensitivity and drought response of trees. Studies have found that dominant trees are more (Mérian and Lebourgeois, 2011; Lebourgeois et al., 2014) or less (Baliva et al., 2024; Zang et al., 2012) sensitive to climate. Lebourgeois et al. (2014) also concluded that tree size does not affect drought response (Lebourgeois et al., 2014). Factors contributing to these varying sensitivities include assimilation, water transport, and carbon allocation, among others (Mérian et al., 2011; Matisons et al., 2017), which may also be influenced by tree species and environmental conditions (Mérian et al., 2011; Lebourgeois et al., 2014). Despite this, the majority of dendrochronological studies predominantly focus on large-diameter trees (Merlin et al., 2015; Hirsch, 2021; Lebourgeois et al., 2014; Castagneri et al., 2012), and dendroclimatological studies that take into account the effects of different diameter classes are minimal.

*Pinus tabuliformis* Carr. and *Quercus variabilis* Blume. are important afforestation species in temperate regions of China, and these species are often cultivated together as mixed forests. The two tree species, *P. tabuliformis* and *Q. variabilis*, have very different water-use strategies, suggesting that they may have different responses to drought (Merlin et al., 2015). *Q. variabilis* is a broad-leaved species with a deep root system (Liu et al., 2018). It exhibits a relatively anisohydric response, reducing its leaf water potential during drought and thus maintaining the drive for water to reach the leaves from the soil (Ferrio et al., 2021). However, it is prone to xylem embolism in this process (Ferrio et al., 2021). In contrast, *P. tabuliformis* is a conifer species with a shallower root system than *Q. variabilis*. It has an isohydric character, i.e., stomata are closed during water deficit to maintain leaf water potential, but this also makes it more susceptible to die from carbon starvation under drought stress (Ferrio et al., 2021). When intermixing *P. tabuliformis* and *Q. variabilis*, there is a co-existence mechanism caused by ecological niche segregation, and this co-existence mechanism is defined by promotion or competition (Del Castillo et al., 2016; Ferrio et al., 2021). Different mixing ratios will likely alter such interactions between the tree species, thereby affecting the response of the tree to drought. Studies on how the stands of these two species, which have different mixing ratios and varying tree diameters, respond to climate change are lacking. To address this gap, this study employed dendrochronology to analyze the responses of dominant, intermediate, and suppressed *P. tabuliformis* and *Q. variabilis* to climate and drought at various mixing ratios: 90% P and 10% Q (P9Q1), 60% P and 40% Q (P6Q4), and 20% P and 80% Q (P2Q8).

Specifically, this study aimed to answer three questions: (1) What is the climate sensitivity and drought response of *P. tabuliformis* and *Q. variabilis*? (2) What are the effects of mixing ratios on climate sensitivity and drought response? (3) How does tree size affect tree response to climate sensitivity and drought, and is this effect consistent across mixing ratios? Three hypotheses were proposed: (1) *P. tabuliformis* is more sensitive to climate and has lower drought tolerance indices than *Q. variabilis*. (2) Both tree species were the least climate sensitive and had the most drought tolerance indices in P6Q4. (3) The effect of individual size on the climate and drought sensitivity of trees is influenced by the mixing ratio.

## 2 Materials and methods

### 2.1 Study area

**Figure 1:**
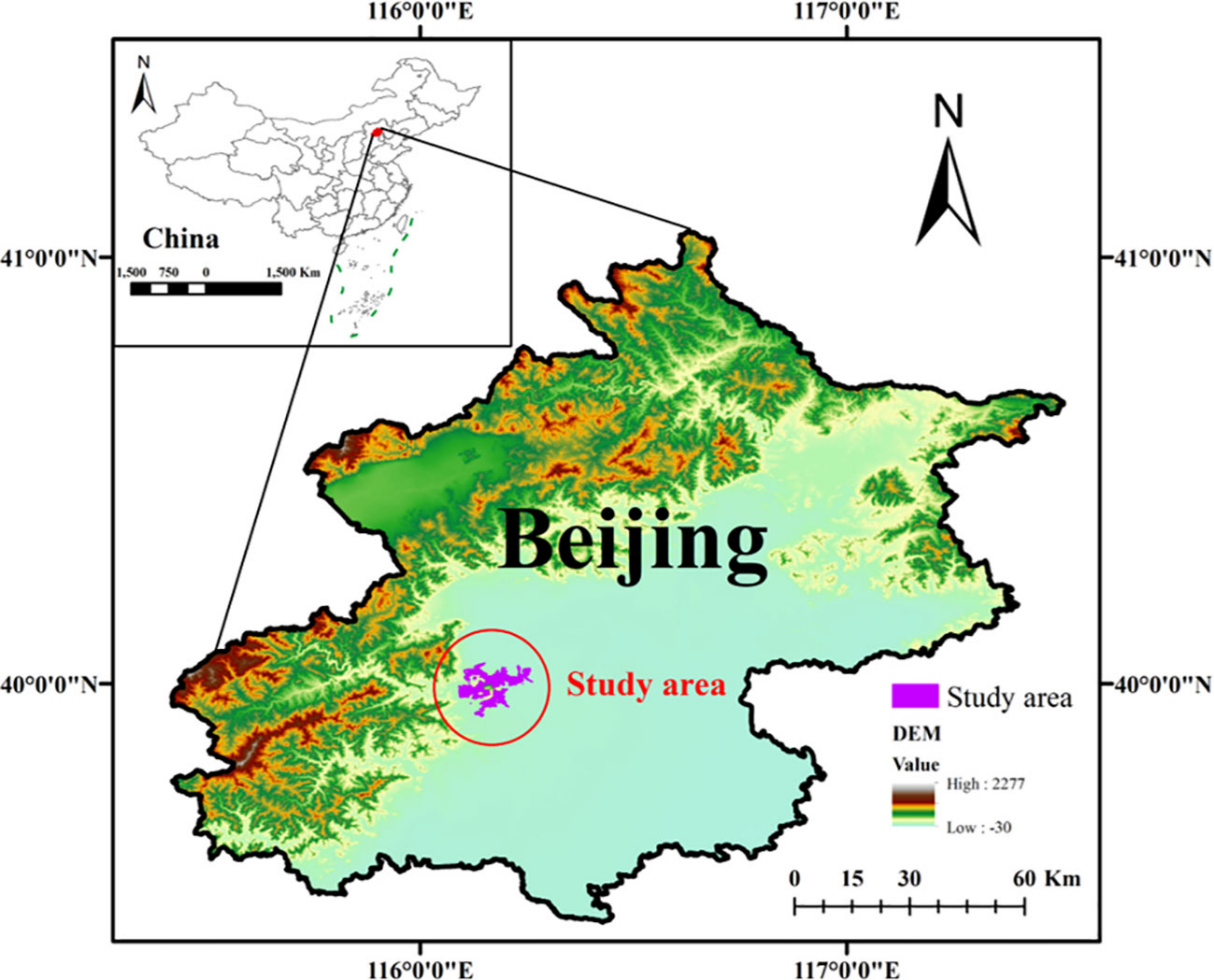
Location of the study area in Beijing, China.

The study area (**Figure 1**) is located in the mountainous region (39°58′18.17″N, 116°11′51.20″E) northwest of Beijing, China, which belongs to the remnants of the Taihang Mountains. The area had an average elevation of 300–400 m (Yu et al., 2023). The climate of the area belongs to the warm temperate semi-humid continental monsoon climate, with high temperatures, rainy summers, and cold, dry winters (Keram et al., 2024). According to the 70-year average data from 1951 to 2020, the average annual temperature of the area is 10.92°C, and the average annual precipitation is 407 mm, mainly concentrated in July and August (**Supplementary Figure A.1**). The soil type in the study area is predominantly mountainous brown soil, characterized by stony and gravelly composition, limited water retention capacity, and a depth of 30 to 50 cm (Liu et al., 2023b). This area has about 90% forest cover, and the main tree species include *Pinus tabuliformis* Carr., *Quercus variabilis* Blume., and *Platycladus orientalis* (L.). Mixed forests of *P. tabuliformis* and *Q. variabilis* are the characteristic forest type of the area, and these forests were primarily established through afforestation efforts in the 1960s and 1970s (Keram et al., 2024).

In July 2021, 12 sample plots were established to investigate stands with three mixed ratios of *P. tabuliformis* (P) and *Q. variabilis* (Q): P9Q1 (90% P and 10% Q), P6Q4 (60% P and 40% Q), and P2Q8 (20% P and 80% Q). Four 25 × 25 m plots were established for each stand type, totaling 12 plots. The plantations on which these plots are located were afforested around 1956 by seedling planting at a spacing of 3 m x 4 m, with an initial density of about 833 plants per hectare. All trees with a diameter ≥ 5 cm were surveyed. The stand information is presented in **Table 1**.

**Table 1:** Stand information.

Mixing ratio	Altitude (m)	Slope aspect	Canopy destiny	Mean DBH (cm) ± SD	Density	Mean H (m) ± SD	Mean crown width (m) ± SD
P9Q1	165	South	0.82	17.7 ± 0.45	824	8.35 ± 0.19	4.56 ± 0.22
P6Q4	183	South	0.85	17.7 ± 1.21	804	8.00 ± 1.02	4.76 ± 0.34
P2Q8	160	South	0.90	18.9 ± 1.58	723	9.66 ± 1.13	5.37 ± 0.35

### 2.2 Tree-ring data

Tree-ring sampling was conducted in the summer of 2023, categorizing *P. tabuliformis* and *Q. variabilis* into three tree size categories: Dominant (D), Intermediate (I), and Suppressed (S). The information on tree size categories can be found in **Table 2**. The sampling strategy included two tree species, three mixing ratios, and three diameter classes, with a total of 290 sampling trees selected. Each tree was cored at 1.3 m above ground in orthogonal directions using a 5.15 mm diameter increment borer (Haglof, Sweden). The 580 collected tree cores were sandpapered (Jing et al., 2022). Tree-ring width (TRW) was measured manually using a Lintab 5 (TSAP; Frank Rinntech, Heidelberg, Germany) with an accuracy of 0.01 mm. COFECHA (V 6.06P) was used to assess the quality and calibration of the cross-dating data. 34 tree-ring cores exhibiting significant bias were excluded, leaving 546 tree-ring cores for chronological analysis. Raw TRW series were detrended using the R package “dplR” (V 1.7.4; Bunn, 2008) to remove age and other factor effects, yielding a standard (STD) chronology. The mean inter-series correlation (r-bar) expressed population signal (EPS) was calculated using COFECHA to verify the quality of the tree-ring chronology series (**Table 2**).

**Table 2:** Sample information and chronological statistics.

Species	Title (DBH range/cm)	Age	n. trees	n. cores	r-bar	EPS
*P.tabuliformis*	P9Q1-D (20.5-30.3)	49(33-59)	22	42	0.14	0.872
	P9Q1-I (15.9-19.5)	45(26-53)	19	36	0.136	0.85
	P9Q1-S (10.9-15.1)	35(21-48)	20	38	0.072	0.745
	P9Q1_total_		61	116	0.112	0.936
	P6Q4-D (20.0-30.0)	48(29-38)	15	30	0.177	0.866
	P6Q4-I (15.9-19.6)	42(27-51)	20	38	0.088	0.785
	P6Q4-S (8.0-15.6)	35(27-58)	16	35	0.04	0.565
	P6Q4_total_		51	103	0.084	0.901
	P2Q8-D (19.5-30.3)	50(45-56)	18	31	0.123	0.812
	P2Q8-I (15.3-19.2)	48(29-59)	17	28	0.061	0.645
	P2Q8-S (11.1-14.1)	40(20-58)	14	27	0.034	0.484
	P2Q8_total_		49	88	0.061	0.851
	*P.tabuliformis*_total_		161	307	0.079	0.962
*Q.variabilis*	P9Q1-D (30.3-37.0)	49(33-59)	8	14	0.121	0.641
	P9Q1-I (21.9-26.8)	45(26-53)	6	12	0.214	0.731
	P9Q1-S (10.0-20.1)	35(21-48)	10	18	0.151	0.661
	P9Q1_total_		24	44	0.139	0.85
	P6Q4-D (29.0-33.7)	48(29-38)	8	16	0.305	0.868
	P6Q4-I (21.3-26.5)	42(27-51)	15	31	0.138	0.812
	P6Q4-S (10.0-20.0)	35(27-58)	17	34	0.05	0.525
	P6Q4_total_		40	81	0.119	0.895
	P2Q8-D (29.0-37.1)	50(45-56)	9	20	0.233	0.859
	P2Q8-I (20.1-27.5)	48(29-59)	22	43	0.192	0.909
	P2Q8-S (10.0-19.9)	40(20-58)	27	51	0.082	0.804
	P2Q8_total_		58	114	0.135	0.944
	*Q.variabilis*_total_		122	239	0.127	0.968

D, I, and S in the table refer to dominant, intermediate, and suppressed trees, respectively.

### 2.3 Meteorological data

The 0.5° × 0.5° resolution gridded dataset of meteorological stations near Xishan National Forest Park (116°11′51.20″E, 39°58′18.17″N) was obtained for 1971–2020 from The Royal Netherlands Meteorological Institute (KNMI) explorer (http://climexp.knmi.nl). The dataset included mean precipitation, mean temperature (Tem), mean minimum temperature (Tmin), mean maximum temperature (Tmax), and the Palmer drought severity index (PDSI). PDSI is a multifactorial climatic indicator that includes precipitation, temperature, evapotranspiration, and other factors. It considers current water supply and demand and the effects of past conditions and duration and effectively reflects regional dry and wet changes. The PDSI better reflects the climatic significance of TRW than a single climate factor (Chen et al., 2023). Given that tree growth can be influenced by the climate of the current and previous years (Fan et al., 2022), the study period in this paper comprised 15 months, from June of the growing season of the previous year to August of the growing season of the current year.

### 2.4 Data analysis

The correlations and significance levels between tree growth and the monthly climate variables for 1971–2020 were evaluated using IBM SPSS 27.0 (IBM Corp, Armonk, NY, USA). *P*< 0.05 was considered to indicate a significant correlation between the variables. The R software (V 4.3.0) package “corrplot” (V 0.92) was used solely for visualizing the correlation results obtained from SPSS 27.0 (IBM Corp, Armonk, NY, USA) and did not involve correlation analysis or significance testing (Zang and Biondi, 2015).

We calculated resistance (Rt), recovery (Rc), and resilience (Rs) indices based on the methodology outlined by Lloret et al. (2011) to evaluate the response of trees to drought and their ability to withstand extreme drought events under climate change:


(1) Rt=Dr/Pre Dr

(2) Rc=Post Dr/Dr

(3) Rs=Post Dr/Pre Dr

where *Dr* represents the TRW of each tree in the drought year, *Pre Dr* represents the average TRW of the two years before each drought event, and *Post Dr* represents the average TRW of the two years after each drought event.

PDSI was employed to identify drought events, with years having an annual average PDSI below −2 classified as drought years (Chen et al., 2023). Consequently, 1972, 1982–1984, 1993, and 1999–2015 were determined to be drought years (**Supplementary Figure A.2**). The year 1972 was excluded because there was only one year of calculated data for the *Pre Dr* before the 1972 drought. Moreover, tree growth dynamics usually differ during the juvenile, adult, and mature stages (Merlin et al., 2015). The selection of the 1993 (transient drought) and 1999–2015 (prolonged drought) ensured that the sampled trees were not in the juvenile stage (**Supplementary Figure A.3**).

A Scheirer-Ray-Hare analysis of variance (ANOVA) was conducted to determine the effects of mixing ratio and tree size and their interaction on the drought resilience parameters. In cases where no significant interaction effect was observed, the main effects of mixing ratio and tree size were tested separately. Subsequent multiple comparisons were performed using the Kruskal–Wallis test to further dissect the variations among different groups.

## 3 Results

### 3.1 Climate sensitivity and drought response of *P. tabuliformis* and *Q. variabilis*

**Figure 2:**
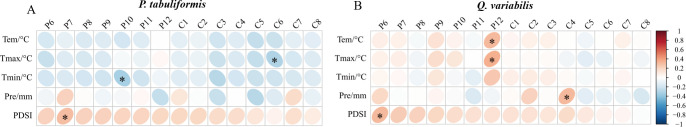
Correlation of radial growth for *P. tabuliformis* **(A)** and *Q. variabilis* **(B)** with monthly climatic factors. The vertical headings in the figure represent meteorological factors: Tem=Temperature, Tmax=Maximum temperature, Tmin=Minimum temperature, Pre=Precipitation, and PDSI=Palmer drought severity index. The horizontal headings denote months, with P6-12 represents June to December of the previous year, and C1-8 refers to January to August of the current year. Grid boxes with black stars indicate statistically significant results (*P*< 0.05).

*P. tabuliformis* radial growth was negatively correlated with temperature, especially with Tmax in June of the current year and Tmin in October of the previous year (**Figure 2A**). *Q. variabilis* radial growth was positively correlated with the temperature in December of the previous year (**Figure 2B**). Both *P. tabuliformis* and *Q. variabilis* radial growth were positively correlated with the PDSI of the previous summer (**Figures 2A, B**). *P. tabuliformis* radial growth was weakly correlated with precipitation (**Figure 2A**). In contrast, *Q. variabilis* radial growth was positively correlated with precipitation in April of the previous year (**Figure 2B**).

**Figure 3:**
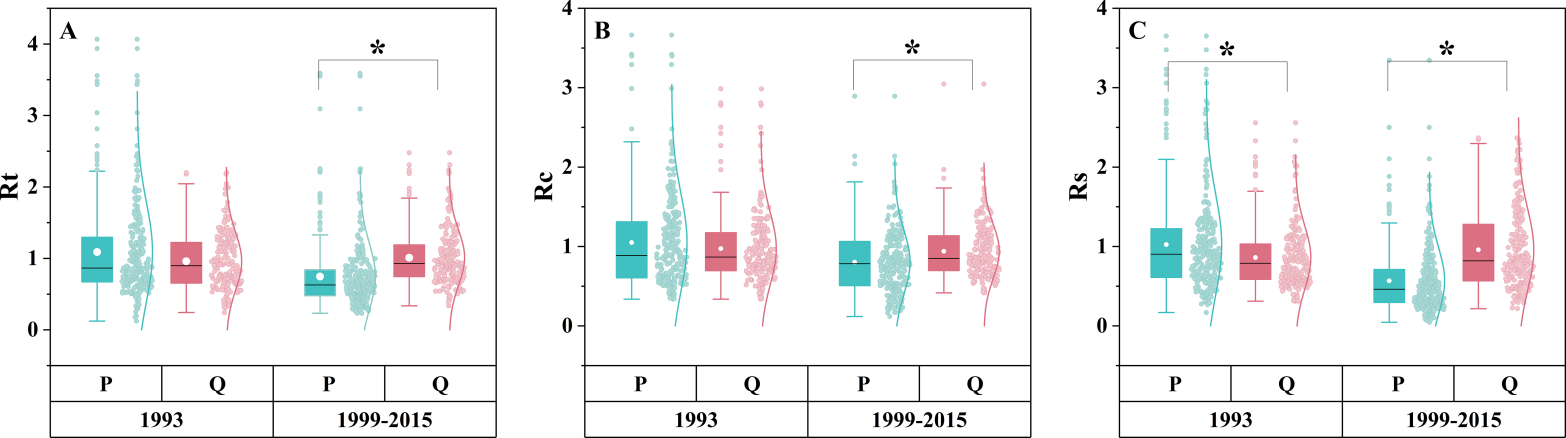
Rt **(A)**, Rc **(B)**, and Rs **(C)** of different tree species during the short-term drought in 1993 and the long-term drought from 1999 to 2015. P in the figure refers to *P. tabuliformis*, and Q refers to *Q. variabilis*. * represents a significant difference between the two groups (*P*< 0.05).

Tree species significantly affected the drought response (**Figures 3A–C**). During the 1993 drought, Rt and Rc were not significantly different between the two species (*P = 0.445*, *P = 0.623*), but Rs was significantly higher in *P. tabuliformis* than in *Q. variabilis* (*P< 0.05*). In contrast, during the prolonged drought from 1999 to 2015, *P. tabuliformis* exhibited significantly lower Rt, Rc, and Rs than *Q. variabilis* (*P< 0.05*), indicating a shift in the drought response depending on the type of drought. Specifically, *P. tabuliformis* performed better than *Q. variabilis* during the short-term drought of 1993, while *Q. variabilis* performed better than *P. tabuliformis* during the long-term drought of 1999–2015.

### 3.2 Effects of mixing ratios on growth–climate relationships and drought response

**Figure 4:**
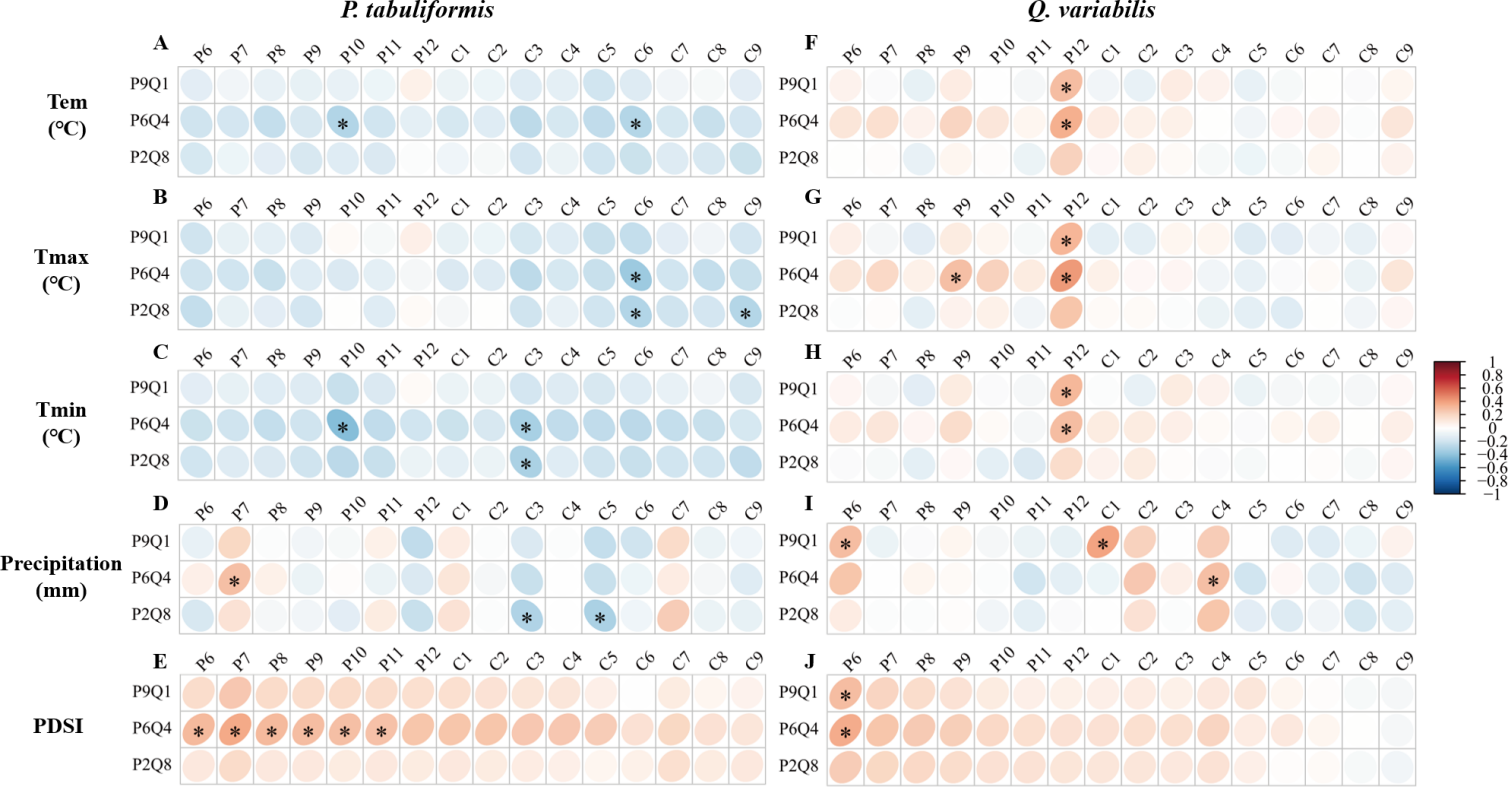
Correlation of radial growth of *P. tabuliformis* and *Q. variabilis* with climatic factors at different mixing ratios. **(A–E)** represent the correlation of radial growth of *P. tabuliformis* with Tem (Temperature), Tmax (Maximum temperature), Tmin (Minimum temperature), Pre (Precipitation), and PDSI (Palmer drought severity index). **(F–J)** indicate the corresponding relationships for *Q. variabilis*. The vertical headings of each subplot show the radial growth of trees at each mixing ratio. The horizontal headings denote monthly climate factors, with P6-12 represents June to December of the previous year, and C1-8 refers to January to August of the current year. Grid boxes with black stars indicate statistically significant results (*P<* 0.05).

The mixing ratios strongly affected the tree growth-climate relationship, as shown in **Figure 4**. None of the correlations of *P. tabuliformis* radial growth with monthly climate factors in P9Q1 reached a significant level (**Figures 4A–E**). In P6Q4, *P. tabuliformis* radial growth showed a significant positive correlation with PDSI in the summer and autumn of the previous year (**Figure 4E**). In P2Q8, *P. tabuliformis* radial growth showed a significant negative correlation with spring precipitation (**Figure 4D**). Additionally, *P. tabuliformis* radial growth in P6Q4 and P2Q8 showed a significant negative correlation with spring Tmin and summer Tmax (**Figures 4B, C**). For *Q. variabilis*, none of the correlations of its radial growth with monthly climate factors in P2Q8 reached a significant level (**Figures 4F–J**). In P9Q1 and P6Q4, *Q. variabilis* radial growth was significantly and positively correlated with the winter temperatures of the previous year (Tem, Tmax, and Tmin) and the PDSI from June of the previous year (**Figures 4F–H, J**).

**Figure 5:**
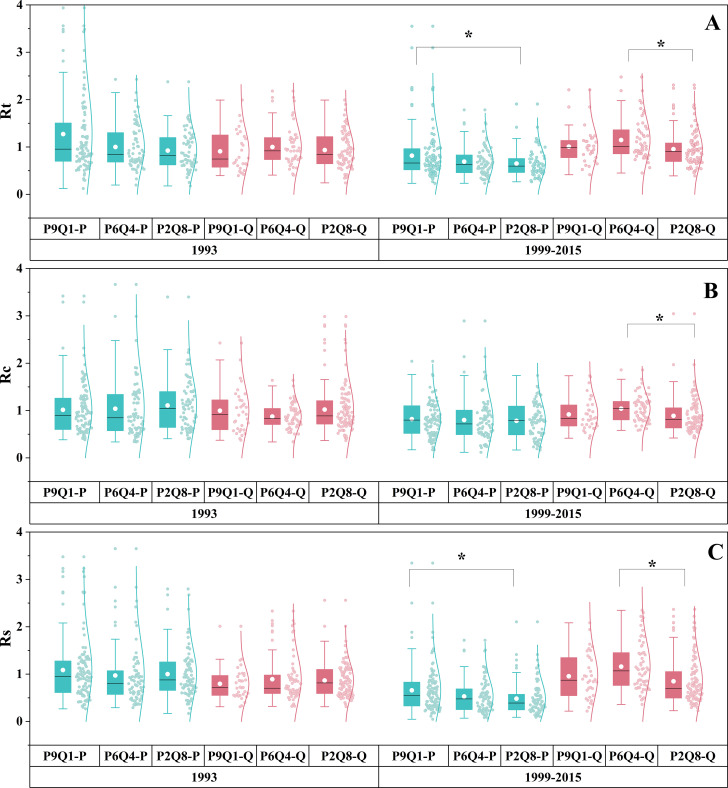
Rt **(A)**, Rc **(B)**, and Rs **(C)** of trees under different mixing ratios during the short-term drought in 1993 and the long-term drought from 1999 to 2015. P in the figure refers to *P. tabuliformis*, and Q refers to *Q. variabilis*. The terms P9Q1 (90% P and 10% Q), P6Q4 (60% P and 40% Q), and P2Q8 (20% P and 80% Q) in the figure refer to different mixing ratios. * indicates a significant difference between the two groups (*P*< 0.05).

The effect of mixing ratios on drought response varied by drought event (**Figure 5**). No significant differences were found in Rt, Rc, and Rs across different mixing ratios during the 1993 drought for either species (*P = 0.091*, *P = 0.206*, *P = 0.130*) (**Figures 5A–C**). The Rc of *P. tabuliformis* remained consistent across the three mixing ratios during the 1999–2015 drought (*P = 0.593*) (**Figure 5B**). Nonetheless, Rt and Rs of *P. tabuliformis* gradually decreased along the sequence P9Q1–P6Q4–P2Q8, with P9Q1 significantly higher than P2Q8 (*P< 0.05*) (**Figures 5A, C**). In contrast to *P. tabuliformis*, *Q. variabilis* exhibited significantly higher Rt, Rc, and Rs than P2Q8 in P6Q4 (**Figures 5A–C**). However, Rt, Rc, and Rs of *Q. variabilis* did not differ between P9Q1 and P6Q4 and P9Q1 and P2Q8 (**Figures 5A–C**).

### 3.3 Effects of tree size on growth–climate relationships and drought response

**Figure 6:**
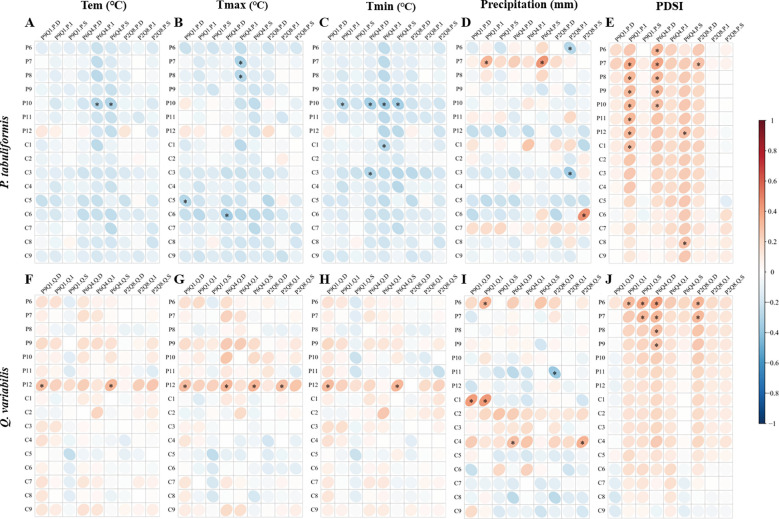
Radial growth-climate relationships of *P. tabuliformis* **(A–E)** and *Q. variabilis* **(F–J)** of different sizes. In each subplot, the horizontal axis titles represent the radial growth of the various groups. P refers to *P. tabuliformis* and Q to *Q. variabilis*. D, I and S refer to dominant, intermediate and suppressed trees, respectively. (For example, P9Q1.P.D refers to the dominant *P. tabuliformis* in the P9Q1). The vertical axis denote monthly climate factors, with P6-12 represents June to December of the previous year, and C1-8 refers to January to August of the current year. Tem, Temperature; Tmax, Maximum temperature; Tmin, Minimum temperature; and PDSI, Palmer drought severity index. Grid boxes with black stars indicate statistically significant results (*P<* 0.05).

The response of trees of different sizes to climatic factors was influenced by mixing ratios (**Figure 6**). In P9Q1, the radial growth of intermediate *P. tabuliformis* exhibited high sensitivity to the PDSI, showing a significant positive correlation with the PDSI from July of the previous year to January of the current year (**Figure 6E**). In contrast, in P6Q4 and P2Q8, the radial growth of dominant *P. tabuliformis* showed the highest sensitivity to PDSI (**Figure 6E**). For *Q. variabilis*, the sensitivity of radial growth to PDSI increased with decreasing tree size in P9Q1, and the radial growth of dominant *Q. variabilis* was insensitive to PDSI (**Figure 6J**). The situation was reversed in P6Q4 and P2Q8, with the radial growth of dominant *Q. variabilis* being more sensitive to PDSI than that of intermediate and suppressed *Q. variabilis* (**Figure 6J**).

According to the ANOVA results, the drought tolerance indices of the two species were influenced by the interaction between the mixing ratio and tree size (**Supplementary Table A.1**). This result emphasizes the importance of considering both factors in drought tolerance index comparisons across different tree sizes.

**Figure 7:**
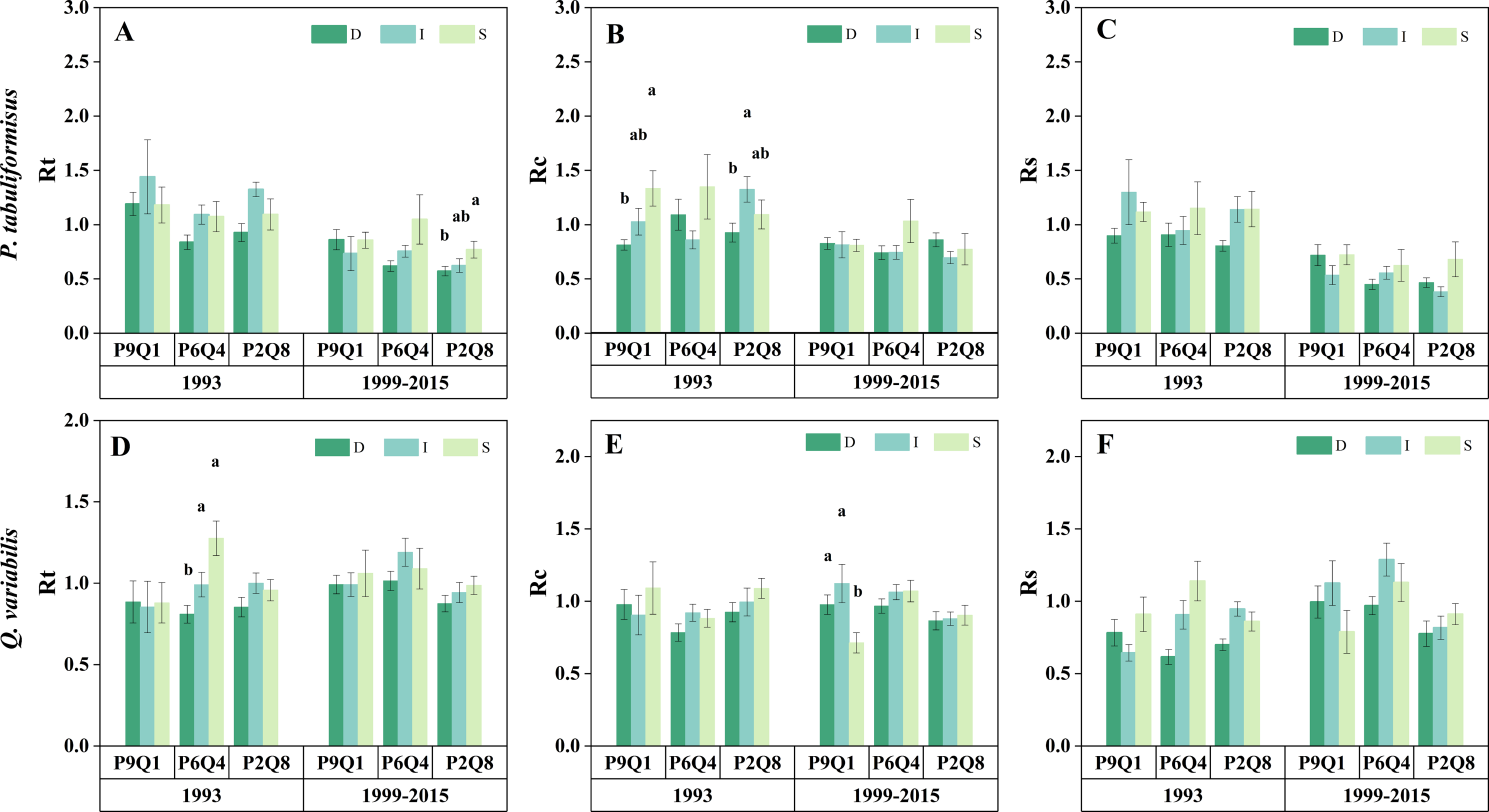
Rt, Rc, and Rs for the dominant (D), intermediate (I), and suppressed (S) trees of *P. tabuliformis* **(A–C)** and *Q. variabilis* **(D–F)** at different mixing ratios during the short-term drought in 1993 and the long-term drought from 1999 to 2015. The terms P9Q1 (90% P and 10% Q), P6Q4 (60% P and 40% Q), and P2Q8 (20% P and 80% Q) in the figure refer to different mixing ratios. Different lowercase letters represent the significant differences between three tree sizes at the *P*< 0.05 level.

The impact of tree size on drought resistance was influenced by the mixing ratio and the type of drought (**Figure 7**). Tree size did not affect the Rs of either tree species in either drought event (**Figures 7C, F**). The Rt of *Q. variabilis* in P6Q4 decreased with an increase in tree size during the 1993 drought (**Figure 7D**). Similarly, the Rt of *P. tabuliformis* in P2Q8 diminished with tree size during the 1999–2015 drought (**Figure 7A**). Additionally, the Rc of *P. tabuliformis* in P1Q9 decreased with increasing tree size in 1993. Rc for *P. tabuliformis* in P2Q8 increased with decreasing tree size, reaching a maximum in intermediate *P. tabuliformis* (**Figure 7B**). However, from 1999 to 2015, the Rc of *Q. variabilis* in P1Q9 increased with tree size, reaching a maximum in intermediate *Q. variabilis* (**Figure 7E**).

## 4 Discussion

### 4.1 *P. tabuliformis* and *Q. variabilis* respond differently to climate and drought events

The radial growth of both *P. tabuliformis* and *Q. variabilis* was positively correlated with the summer PDSI of the previous year (**Figures 2A, B**), suggesting that drought events occurring in the previous summer reduce tree carbohydrate storage and thus affect radial growth in the coming year (Zhang et al., 2016a; Fuchs et al., 2021). The radial growth of *P. tabuliformis* and *Q. variabilis* responded differently to temperature and precipitation, which is consistent with our first hypothesis. *P. tabuliformis* radial growth was adversely affected by elevated Tmax in summer and Tmin in the previous autumn (**Figure 2A**). High summer temperatures induce stomatal closure in plant leaves, thus decreasing the production of photosynthetic assimilates (Barber et al., 2000; Jiao et al., 2016). High temperatures also increase the embolism in xylem conduits, significantly limiting water uptake efficiency (Deslauriers et al., 2014). High minimum temperatures in the previous autumn were also associated with lower *P. tabuliformis* growth. High nighttime minimum temperatures increase respiration, increasing nighttime carbon loss and harming tree growth in the following year (Zhang et al., 2016b). In contrast to *P. tabuliformis*, *Q. variabilis* radial growth did not negatively correlate with summer temperature (**Figure 2B**). This result suggests that *Q. variabilis* is tolerant to high summer temperatures at low altitudes (mean altitude 300–400 m), at least when annual precipitation is above 600 mm. *Q. variabilis* radial growth was positively correlated with winter temperature (**Figure 2B**), whereas no significant correlation was observed between *P. tabuliformis* radial growth and winter temperature (**Figure 2A**). This result also suggests that *Q. variabilis* depends more on warmer winter temperatures to survive the cold season than *P. tabuliformis*. Furthermore, the lack of a significant correlation between *P. tabuliformis* radial growth and precipitation indicates that precipitation has little effect on *P. tabuliformis* monthly and that high-temperature-induced drought is the climatic factor limiting growth in *P. tabuliformis* (**Figure 2A**). Unlike *P. tabuliformis*, *Q. variabilis* radial growth was significantly influenced by spring precipitation (**Figure 2B**). The high sensitivity of oak species to spring moisture availability has been confirmed in several studies (Merlin et al., 2015; Zang et al., 2012; Morán-López et al., 2014). This is because early xylem vessels in oak species are formed using stored photosynthetic products from the previous growing season (Gil-Pelegrin et al., 2004). The large diameter of the vessels formed during this period and the high efficiency of water transport increase the risk of embolism. Spring precipitation alleviates the impeded cell enlargement caused by the lack of water in the tree (Tardif and Conciatori, 2006).

The differences between species in their response to drought were mainly observed during the prolonged drought (1999–2015) (**Figures 3A–C**). Rt, Rc, and Rs of *Q. variabilis* were all greater than those of *P. tabuliformis* (**Figures 3A–C**), indicating that *Q. variabilis* has a high resistance to water stress and fast recovery. Our findings confirm previous studies that have reported a greater sensitivity of pine species to drought, indicating a higher vulnerability of pine species than oak species (Bello et al., 2019; Steckel et al., 2020). Interestingly, the responses of the two species to drought varied depending on the duration of the drought. Unlike prolonged drought, there was no difference in Rt and Rc between *P. tabuliformis* and *Q. variabilis* in short-term drought. Still, the Rs of *P. tabuliformis* were significantly greater than that of *Q. variabilis* (**Figures 3A–C**). In conclusion, unlike our hypothesis, *Q. variabilis* is more resistant than *P. tabuliformis* under long-term droughts, while the opposite is true under short-term droughts. The observed differences in the drought response between *P. tabuliformis* and *Q. variabilis* were attributed to the distinct water utilization strategies employed by the two species under drought conditions. Isohydric species, such as *P. tabuliformis*, close their stomata to avoid water loss during drought. Conversely, anisohydric species, such as *Q. variabilis*, continue transpiring until water resources are depleted (Zang et al., 2012). Therefore, *P. tabuliformis* performs better than *Q. variabilis* during short-term droughts. However, long-term stomata closure by isohydric species during prolonged drought severely limits access to water and carbon, leading to slower growth and possibly death (Liang et al., 2021). Anisohydric species may be better able to adapt by tweaking transpiration and other physiological mechanisms to delay water loss while maintaining relatively high growth rates. Therefore, *Q. variabilis* outperforms *P. tabuliformis* during long-term drought (Liu et al., 2023a).

### 4.2 Mixing ratios affect growth–climate relationships and drought response

A large number of previous studies on the climate sensitivity of mixed pine-oak forests have focused on pure versus mixed forests, suggesting that mixed forests reduce (Liu et al., 2022; Steckel et al., 2020; Thurm et al., 2016; Cao et al., 2022) or increase (Fuchs et al., 2021; Nothdurft and Engel, 2020) the climate sensitivity of trees. However, little attention has been paid to whether different proportions of pine-oak mixtures may affect growth–climate responses differently. We analyzed the growth–climate relationships of trees with varying mixing ratios and found that the effects of mixing ratios on tree growth–climate relationships varied by species. For *P. tabuliformis*, its growth in P6Q4 and P2Q8 was significantly negatively correlated with spring and summer temperatures (**Figures 4A–C**), suggesting that high spring and summer temperatures were detrimental to *P. tabuliformis* in P6Q4 and P2Q8. The effects of high temperature-induced drought on tree growth during the spring and summer seasons are common worldwide (Schwarz et al., 2020; Schwab et al., 2018). At the same time, P9Q1 mitigated the negative effect of high temperatures on the radial growth of *P. tabuliformis* during the spring and summer seasons (**Figures 4A–C**), which might be related to the fact that *P. tabuliformis* in P9Q1 was subjected to minimal interspecific competition from *Q. variabilis*. *P. tabuliformis* radial growth in P2Q8 was negatively correlated with spring precipitation (**Figure 4D**). Spring is the most critical period for the initiation of cambium activities. Spring precipitation is crucial in replenishing soil water availability depleted by temperature rise, supporting root development, and facilitating water and nutrient uptake (Zhang et al., 2021). Therefore, an adequate water supply in spring is favorable for xylem development and cell production and promotes cell division in the cambium (Dawadi et al., 2013). However, our results indicate that *P. tabuliformis* radial growth in P2Q8 negatively correlates with spring precipitation. This phenomenon may be because large stands of *Q. variabilis* in P2Q8 consume significant amounts of water in spring, reducing the available water for *P. tabuliformis* during this critical period and limiting its growth. In addition, among the three mixing ratios, only the radial growth of *P. tabuliformis* in P6Q4 showed a strong correlation with PDSI (**Figure 4E**), suggesting that P6Q4 forced *P. tabuliformis* to suffer severe drought stress. For *Q. variabilis*, P2Q8 mitigated the climate sensitivity of the radial growth of *Q. variabilis*. First, the radial growth of *Q. variabilis* in P9Q1 and P6Q4 positively correlates with winter temperatures, while in P2Q8, the radial growth of *Q. variabilis* is not significantly correlated with winter temperatures (**Figures 4F–H**). This suggests that *Q. variabilis* in P2Q8 has more nutrient reserves, relatively greater vigor, and higher resistance to low temperatures than the other mixing ratios, which may be related to the high productivity of cold-resistant substances (Matisons et al., 2017). This implies that winter warming in Beijing promotes the growth of *Q. variabilis* in P9Q1 and P6Q4, while it has little effect on *Q. variabilis* in P2Q8. Second, the radial growth of *Q. variabilis* in P9Q1 and P6Q4 was significantly positively correlated with PDSI, while P2Q8 was not (**Figure 4J**). Sufficient water in the previous summer accumulated the carbon matter needed for tree growth the following year and promoted root growth (Galiano et al., 2011). P2Q8 mitigated the effects of high temperatures in the previous summer on the radial growth of *Q. variabilis*. This may be related to the high number of broad-leaved species in P2Q8, the relatively wet environment within the forest, and the low surface runoff, which facilitates water infiltration and allows the growth of trees to maximize the use of water resources. In conclusion, a very interesting result is that the radial growth of *P. tabuliformis* has the weakest relationship with climate factors in P9Q1. In contrast, the radial growth of *Q. variabilis* has the weakest relationship with climate factors in P2Q8, which is contrary to our second hypothesis. This suggests that, whether it is *P. tabuliformis* or *Q. variabilis*, they are both least affected by climate in stands that are least exposed to interspecific effects from other species.

Analyzing the effect of mixing ratios on drought response revealed that *Q. variabilis* showed higher Rt, Rc, and Rs in P6Q4 compared to P2Q8 and P9Q1 under prolonged drought conditions. (**Figures 5D–F**), confirming our second hypothesis. However, no differences were found in Rt, Rc, and Rs between P9Q1 and P2Q8 for *Q. variabilis* (**Figures 5D–F**). These results suggest that mixed plantings with appropriate proportions of *P. tabuliformis* enhance the resistance, recovery, and resilience of *Q. variabilis*. However, the promotion of *Q. variabilis* weakens if the proportion of *P. tabuliformis* is too high. The hypothesis of complementary effects in mixed forests suggests that the presence of one species in a mixed forest may improve the environmental conditions of another species. Subsequently, Pretzsch et al. (2013) demonstrated the existence of the complementary effects hypothesis in mixed pine-oak forests. Specifically, *Q. variabilis* in mixed stands may benefit from the more conservative stress response strategies of *P. tabuliformis* under drought stress, resulting in improved water availability (Pretzsch et al., 2013). This implies that water use by *Q. variabilis* in mixed stands should increase with increasing proportions of *P. tabuliformis*. This explains why the response of *Q. variabilis* to drought was better in P6Q4 than in P2Q8. However, P9Q1 did not further contribute to the drought tolerance indices of *Q. variabilis* relative to P6Q4 and P2Q8. We speculate that this may be due to the predominance of conifers in P9Q1, which had the lowest water breaks, the relatively poor water-holding capacity of dead wood, and the lowest soil water-holding capacity (Bai, 2006; Lu et al., 2023). Additionally, *Q. variabilis* undergoes hydraulic lift under drought conditions (Caldwell et al., 1998; Jonard et al., 2011; Zapater et al., 2011; Hafner et al., 2017), transferring deep water to shallow layers, which further reduces the amount of water available for *Q. variabilis*. The complementary effects of *P. tabuliformis* on the water use of *Q. variabilis* and the reduction in water use due to the lowest soil water-holding capacity of the stand counterbalance each other. This resulted in no differences in the resistance and resilience of *Q. variabilis* in P9Q1 and P2Q8. For *P. tabuliformis*, resistance and resilience decreased with increasing *Q. variabilis* proportions in this study (**Figures 5D–F**). This contradicts our second hypothesis. This phenomenon may be explained by interspecific competition, where the strategy of *Q. variabilis* to use more water during drought stress may reduce the amount of water available to *P. tabuliformis* in the mixture, leading to increased rather than complementary interspecific competition, which favors the more competitive species (*Q. variabilis*) to the detriment of the other species (*P. tabuliformis*) (Bello et al., 2019). Our second hypothesis that P6Q4 would improve drought tolerance in *P. tabuliformis* and *Q. variabilis* was confirmed only in *Q. variabilis*.

### 4.3 The response of growth to climate and drought is affected by tree size

Consistent with our hypothesis, there was variation in growth-climate relationships between individual sizes, which was inconsistent across mixing ratios (**Figure 6**). This implies that there are also different growth strategies among trees of various size classes, even though they grow in a familiar environment with common biophysical and climatic conditions (De Luis et al., 2009). Considering the variation in water sensitivity by tree size, the literature gives conflicting results. Jacquart et al. (1992) reported that smaller trees experience more significant water stress due to greater root competition for soil moisture (Jacquart et al., 1992; Abrams and Mostoller, 1995), resulting in smaller trees being more sensitive to climatic factors. On the other hand, Carrer and Urbinati (2004) and Trouillier et al. (2019) reported that larger trees are more sensitive than smaller trees (Carrer and Urbinati, 2004; Trouillier et al., 2019). Because dominant trees typically have higher heights, their increased sensitivity to drought stress has been linked to the “hydraulic limitation hypothesis” (Ryan and Yoder, 1997), as the hypothesis suggests that hydraulic resistance increases with height (Trouillier et al., 2019). In our study, the effect of tree size on climate response varied according to mixing ratios. In P6Q4 and P2Q8, the radial growth of dominant *P. tabuliformis* and *Q. variabilis* had the highest sensitivity to water stress due to high summer temperatures (**Figure 6**). This result contradicts the idea of root competition described above. This may be related to the hydraulic transport limitation hypothesis and the idea that intermediate and suppressed trees gain from a stable microclimate environment. Unlike P6Q4 and P2Q8, however, the radial growth of intermediate *P. tabuliformis* and suppressed *Q. variabilis* were the most sensitive to water stress in P9Q1 (**Figure 6**). This may be related to soil moisture content (Mérian et al., 2011) and litter nutrient content moderated differences in drought sensitivity between tree sizes between stands. The physiological and ecological reasons for our observed differences cannot be fully explained due to the lack of relevant references. However, we focus on conveying that climate change may affect trees differently and alter the stand structure in the coming decades due to the different climate sensitivity of trees of various sizes (Merlin et al., 2015).

The results of the study on the impact of individual size on the drought sensitivity of trees showed that in terms of *P. tabuliformis*, the tree that was suppressed during a short-term drought had the most recovery in P9Q1 (**Figure 7B**). This result was probably due to its ability to use limited resources more efficiently in an environment with less competitive pressure. There was no significant difference between the drought tolerance indices of different sizes of *P. tabuliformis* in P9Q1 under prolonged drought conditions, suggesting they have similar adaptations to drought stress (**Figures 7A–C**). The dominant *P. tabuliformis* showed the lowest recovery in short-term drought and resistance in long-term drought in P2Q8 (**Figures 7A, B**). This suggests that the competitiveness of *P. tabuliformis* was significantly limited in environments with higher proportions of *Q. variabilis*. Limited water resources were insufficient to satisfy the dominant *P. tabuliformis* (Bennett et al., 2015). For *Q. variabilis*, suppressed trees under long-term drought in P9Q1 had the lowest recovery rate, indicating a reliance on competitive positioning for water. In P6Q4, short-term drought affected dominant trees more, while in P2Q8, size did not influence drought tolerance. These results suggest that the effect of individual size on tree drought resilience is complex and moderated by the species stand environment. In conclusion, this result confirms the third hypothesis that the mixing ratio influences the effect of individual size on the drought sensitivity of trees. Therefore, we emphasize that an integrated consideration of mixing ratios and stand structure (diameter class regulation) is essential for accurately understanding the impact of drought on forest ecosystems and developing specific forest management strategies.

### 4.4 Forest management and adaptation to drought

For forest managers, this study provides important references and insights for choosing appropriate mixing ratios, optimizing forest stand structures, and developing drought-resilient management strategies. Current climate projections indicate climate change will increase drought events in temperate regions (Charlet De Sauvage et al., 2023). Thus, forest policies encourage more tree species in diverse forests to increase productivity and reduce vulnerability to stress and disturbances (Grossiord, 2020). However, how to choose the proper mixing ratio to maximize these benefits is often overlooked. This study found that mixing ratios significantly impacted the climate and drought responses of trees. High proportions of *Q. variabilis* should be avoided when establishing *P. tabuliformis*–*Q. variabilis* mixed forests because they reduce the drought tolerance indices of both *P. tabuliformis* and *Q. variabilis*, which is detrimental to the stability of the entire stand. In mixed forests of *Pinus tabuliformis* and *Quercus variabilis* in semi-arid regions, maintaining a relatively balanced configuration of the two species may be an effective management strategy, as it has been demonstrated to significantly enhance the drought resilience of *Q. variabilis* without adversely affecting the drought resilience of *P. tabuliformis*. This mixing ratio has a clear advantage in coping with drought events. It is essential for maintaining the overall productivity and stability of the stand during drought events in the long run. Additionally, trees of different size classes have different water needs and access capacities in a stand. Tree size affects tree response to climate and drought; mixing ratios modulate this effect. Matching an appropriate stand structure with specific mix proportions is critical in forest management to help mitigate drought-induced declines in stand productivity.

## 5 Conclusion

This study emphasizes the critical role of mixing ratios and tree size in plant adaptation to climate change and drought events. The findings revealed that the response of trees to drought varied with drought duration. *P. tabuliformis* performed better during the 1993 short-term drought, whereas *Q. variabilis* showed better during the 1999 to 2015 long-term drought. P6Q4 increased the sensitivity of *P. tabuliformis* radial growth to PDSI compared to the other two mixing ratios. The mixing ratio has a complex impact on tree drought resilience, with the effects becoming more pronounced during longer droughts. Differences in soil water availability enabled *Q. variabilis* to benefit from hybridization with *P. tabuliformis*. However, the proportion of *P. tabuliformis* must be carefully controlled; too high or too low a proportion may reduce the promoting effect of the mixture on *Q. variabilis*. During a drought, *Q. variabilis*, a competitive dominant tree species, stresses *P. tabuliformis* with water. Reducing the proportion of *Q. variabilis* benefits the survival of *P. tabuliformis*. These findings indicate that properly adjusting the tree species mixing ratios in arid conditions is essential for maintaining ecological balance and forest ecosystems stability. The adaptability and resilience of forests to extreme climatic conditions can be enhanced through scientific management of the ratio between different tree species. Furthermore, we demonstrated that tree of various sizes respond differently to climate and drought sensitivity. Therefore, future forest management should include the development of appropriate management policies for trees of different sizes.

## Data Availability

The raw data supporting the conclusions of this article will be made available by the authors, without undue reservation.
