# Supplementary material for: Combined effects of mixing ratios and tree size: how do mixed forests respond to climate and drought events?
Source: Front Plant Sci. 2024 Oct 10;15:1477640. doi: 10.3389/fpls.2024.1477640 (PMC11499116; doi:10.3389/fpls.2024.1477640)
Supplement: Supplementary file 1 [file DataSheet1.docx]

Supplementary Material

# Supplementary Figures and Tables

Table A.1 Scheirer-Ray-Hare analysis of variance (ANOVA) regarding the effects of stand composition (SC) and tree size (TS), and their interactions (SC ×  TS) on the drought resilience parameters.

| **Species** | **Factors** |  | **Rt** | | **Rc** | | **Rs** | |
| --- | --- | --- | --- | --- | --- | --- | --- | --- |
|  |  |  | **1993** | **1999-2015** | **1993** | **1999-2015** | **1993** | **1999-2015** |
| *P. tabuliformis* | SC | F | 5.423 | 2.92 | 0.207 | 0.448 | 0.59 | 3.027 |
|  |  | P | **0.005** | 0.056 | 0.813 | 0.639 | 0.555 | **0.05** |
|  | TS | F | 0.841 | 3.964 | 4.762 | 1.595 | 5.119 | 3.33 |
|  |  | P | 0.433 | **0.02** | **0.009** | 0.205 | **0.007** | **0.037** |
|  | SC×TS | F | 1.291 | 1.597 | 3.358 | 1.634 | 0.955 | 1.4 |
|  |  | P | 0.274 | 0.176 | **0.011** | 0.166 | 0.433 | 0.234 |
| *Q. variabilis* | SC | F | 1.625 | 3.234 | 1.756 | 3.731 | 0.827 | 6.385 |
|  |  | P | 0.2 | **0.042** | 0.176 | **0.026** | 0.439 | **0.002** |
|  | TS | F | 2.73 | 0.76 | 0.974 | 2.136 | 6.047 | 1.649 |
|  |  | P | 0.068 | 0.469 | 0.38 | 0.121 | **0.003** | 0.195 |
|  | SC×TS | F | 1.848 | 0.369 | 0.319 | 2.322 | 2.686 | 1.535 |
|  |  | P | 0.121 | 0.83 | 0.865 | 0.058 | **0.033** | 0.194 |

Significant effects are presented in bold (*P* ≤ 0.05).


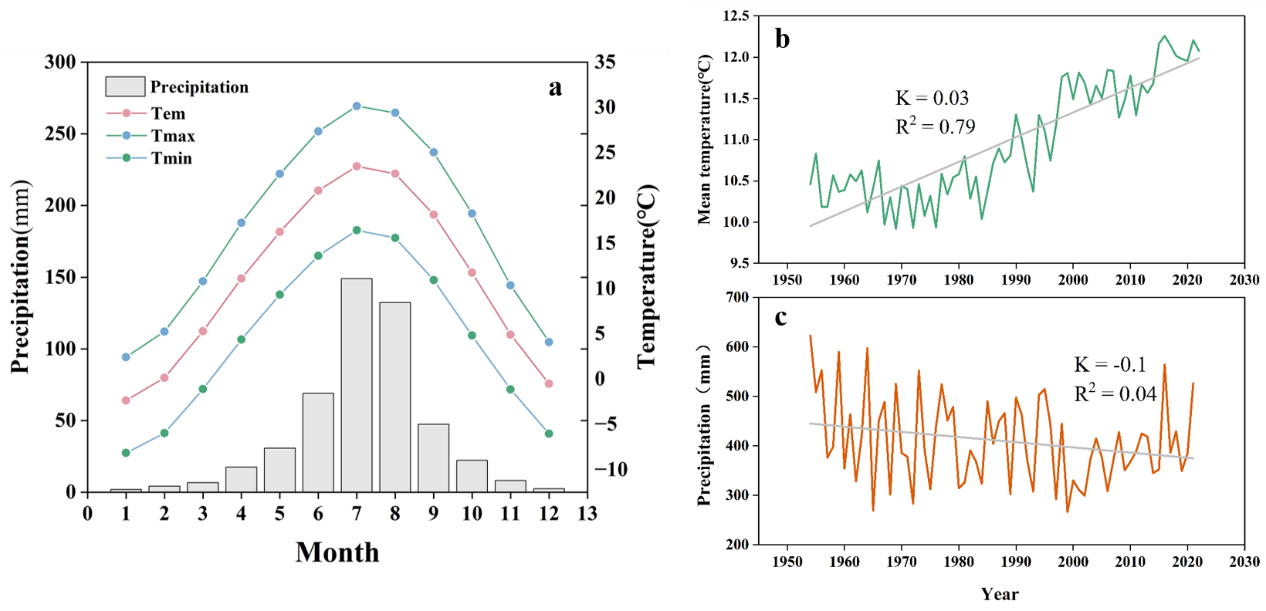


Figure. A.1 Climatic conditions in the study area. a. Total monthly precipitation and average monthly temperature in the study area. Tem is the mean temperature. Tmax is the maximum temperature. Tmin is the lowest temperature. b. Mean temperature trends in the study area since 1950. c. Trends in precipitation in the study area since 1950.


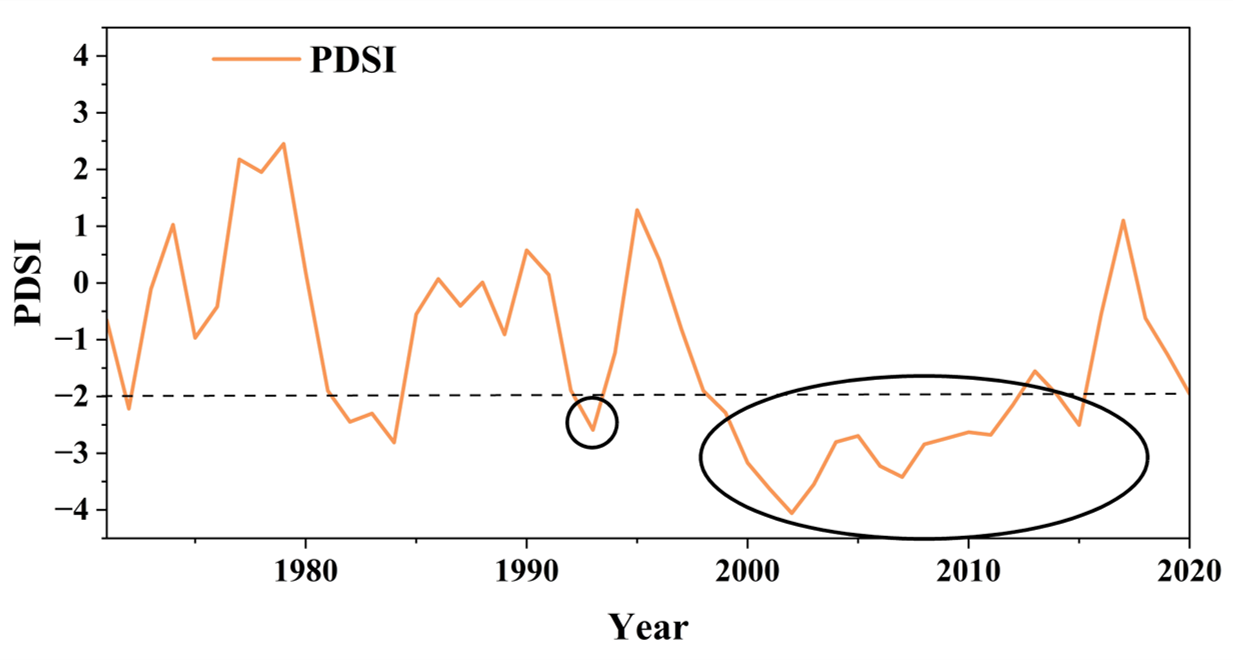


Figure. A.2 The PDSI was calculated for 1971 – 2020. The bold circle indicates selected drought events and the horizontal dotted line is the threshold of –2 below which a drought was considered severe. The two circles are for two drought events in 1993 and 1999 – 2015.


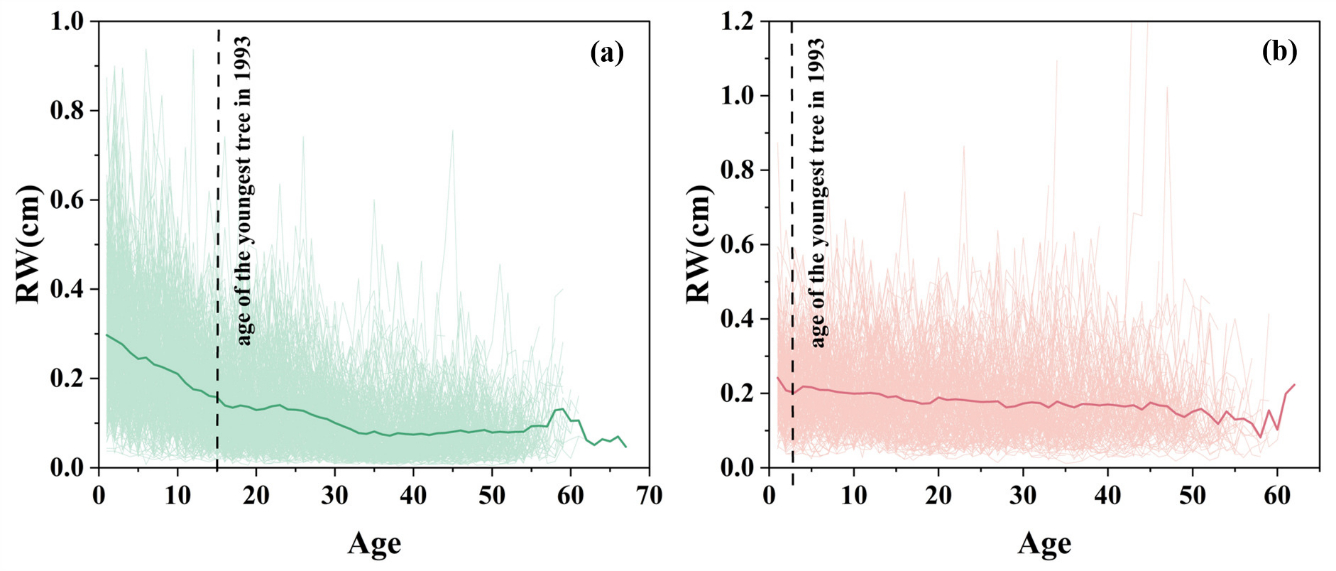


Figure. A.3 Raw tree-ring width (RW) growth series for *P. tabuliformis* (a) and *Q. variabilis* (b) based on age. Shading indicates the overlap of the annual growth series for each tree, and the thick line symbolizes the mean value for all trees. The vertical dotted line indicates the age in 1993 (the youngest drought studied here) of the youngest tree sampled.
